# Epileptic Seizures Detection in EEG Signals Using Fusion Handcrafted and Deep Learning Features

**DOI:** 10.3390/s21227710

**Published:** 2021-11-19

**Authors:** Anis Malekzadeh, Assef Zare, Mahdi Yaghoobi, Hamid-Reza Kobravi, Roohallah Alizadehsani

**Affiliations:** 1 Department of Electrical Engineering, Gonabad Branch, Islamic Azad University, Gonabad 6518115743, Iran; anismalekzade@yahoo.com; 2 Department of Electrical Engineering, Mashhad Branch, Islamic Azad University, Mashhad 9187147578, Iran; yaghoobi@mshdiau.ac.ir (M.Y.); hkobravi@mshdiau.ac.ir (H.-R.K.); 3 Institute for Intelligent Systems Research and Innovation (IISRI), Deakin University, Waurn Ponds, VIC 3216, Australia; r.alizadehsani@deakin.edu.au

**Keywords:** epileptic seizures, EEG, diagnosis, TQWT, nonlinear features, CNN–RNN

## Abstract

Epilepsy is a brain disorder disease that affects people’s quality of life. Electroencephalography (EEG) signals are used to diagnose epileptic seizures. This paper provides a computer-aided diagnosis system (CADS) for the automatic diagnosis of epileptic seizures in EEG signals. The proposed method consists of three steps, including preprocessing, feature extraction, and classification. In order to perform the simulations, the Bonn and Freiburg datasets are used. Firstly, we used a band-pass filter with 0.5–40 Hz cut-off frequency for removal artifacts of the EEG datasets. Tunable-Q Wavelet Transform (TQWT) is used for EEG signal decomposition. In the second step, various linear and nonlinear features are extracted from TQWT sub-bands. In this step, various statistical, frequency, and nonlinear features are extracted from the sub-bands. The nonlinear features used are based on fractal dimensions (FDs) and entropy theories. In the classification step, different approaches based on conventional machine learning (ML) and deep learning (DL) are discussed. In this step, a CNN–RNN-based DL method with the number of layers proposed is applied. The extracted features have been fed to the input of the proposed CNN–RNN model, and satisfactory results have been reported. In the classification step, the K-fold cross-validation with k = 10 is employed to demonstrate the effectiveness of the proposed CNN–RNN classification procedure. The results revealed that the proposed CNN–RNN method for Bonn and Freiburg datasets achieved an accuracy of 99.71% and 99.13%, respectively.

## 1. Introduction

Epilepsy is a noncontagious disease and one of the most prominent brain disorders. About 1% of the world’s population has been diagnosed with epilepsy [1]. Patients with epileptic seizures suffer from some temporary electric disorders [1,2,3]. About 20–30 percent of the patients diagnosed with epilepsy experience one or more strokes in a month [4,5,6]. In the epileptic seizures period, physical damages might even cause the death of the patient. The patients also suffer from lack of a good social position and experience some severe mental disorders [4,5,6].

In 2017, the International League Against Epilepsy (ILAE) presented a new classification of the epileptic seizure types: focal epilepsy, generalized epilepsy, and epilepsy with unknown symptoms [7]. In this classification, some detailed and precise information about each of the epileptic seizure types, including the types and the brain areas experiencing convulsion, are presented [7]. The early diagnosis of epileptic seizures has enormous importance and will prevent the disease progression significantly.

Many screening methods to diagnose epilepsy have been proposed until now, and the neuroimaging modalities have gained much attention from the specialized Specialist doctors [8]. Basically, the neuroimaging modalities in the diagnosis process of epileptic seizures include structural and functional methods. In the neuroimaging modalities, an epileptic seizure diagnosis based on EEG signals has remarkable popularity. EEG signal recording includes scalp EEG (sEEG) and intracranial EEG (IEEG) modalities [9]. EEG modalities include essential information from the functions of the brain in the epileptic seizures period. In comparison with other neuroimaging modalities, some benefits of EEG are a lower cost, the easiness of carrying, and suitable performance in epileptic seizure detection [9]. To diagnose epileptic seizures, doctors need to have a long record of the patient’s EEG signals. The EEG signals also usually have many various channels and artifacts, which cause some difficulties and challenges for doctors in the epileptic seizures diagnosis process [9,10].

To address these challenges, using CADS based on artificial intelligence (AI) can help to improve the speed and accuracy of the epilepsy diagnosis process [11,12,13]. AI-based CADS include ML and DL methods [14,15,16,17]. The most significant difference between CADS based on ML and DL is in the feature extraction step [9]. In CDAS based on ML, the most important feature extraction techniques include the time domain, frequency, and nonlinear features [18]. Choosing different feature extraction algorithms together to reach a high diagnosis accuracy demands a fair amount of knowledge in the field of ML [19,20].

On the other hand, the feature extraction and selection steps in CADS based on DL will be implemented on the deep layers. Many research projects are being conducted in the field of epileptic seizures diagnosis using DL and ML techniques [21,22,23,24,25,26,27,28,29,30,31,32,33,34,35,36,37,38,39,40,41,42,43,44,45,46,47,48,49,50,51,52,53,54,55,56,57,58,59,60,61,62,63,64,65,66,67,68,69,70,71,72,73,74,75,76]. The purpose of these papers is to reach an authentic and accurate epileptic seizures diagnosis using EEG signals.

**Figure 1:**
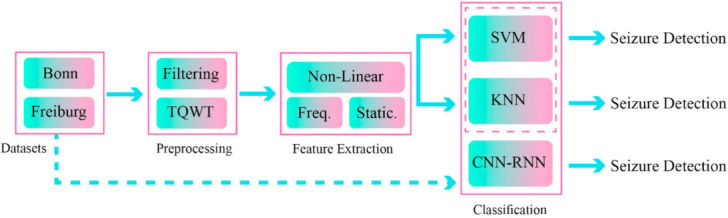
Proposed method for epileptic seizure detection.

One recently developed AI field in epileptic seizures detection uses feature fusion techniques [77,78]. In these methods, a combination of features from different domains will improve the functionality and accuracy of the disease diagnosis process [77,78]. In this work, a novel epileptic seizure diagnosis method using a combination of handcrafted features and DL has been proposed; the summary of its steps is shown in Figure 1.

The proposed method includes the dataset, preprocessing, feature extraction, and classification steps. The two different datasets of Bonn [79] and Freiburg [80] were used to implement the proposed method. In the preprocessing step, the TQWT was used in EEG signal decomposition of different sub-bands.

Three variables are used for adjusting and reducing the search space of filter banks. The three important parameters of TQWT are the Q-factor, redundancy (*r*), and the number of sub-bands (*J*) [81]. The parameters *Q* = 1, *r* = 3, and *J* = 8 were chosen in this paper, similar to Reference [82]. After EEG signal decomposition using TQWT, various statistical, frequency, and nonlinear features are extracted. The EEG signals have a chaotic and nonlinear nature. Related works showed that nonlinear feature extraction methods play a significant role in improving the functionality and accuracy of the epileptic seizure diagnosis using EEG signals [23,24,25,26,27,28,29,30,31,32,33,34,35,36,37,38,39,40]. The most important nonlinear feature extraction methods from EEG signals include various types of entropies [83], FDs [84], graphs [85], the largest Lyapunov exponent (LLE) [86], and correlation coefficients (CC) [87]. In this step, various statistical, frequency, and nonlinear features are extracted in the TQWT sub-bands.

In this paper, a novel class of entropy and fractal theory-based features was used. The combination of this class of handcrafted features was used in this paper for the first time as the first innovation. In this section, feature extraction algorithms were chosen and combined based on exploring other research papers and, also, their epileptic seizure diagnosis functionality. Fractal-based nonlinear features include Higuchi [88], Katz [88], Petrosian [88], and the detrended fluctuation analysis (DFA) [89,90]. Entropy-based feature extraction techniques also include Shannon [91,92,93], Log-Energy [93], spectral [94], Sample [95], permutation [96], Fuzzy [97], refined composite multiscale fuzzy [98], graph [99], Permutation Rényi [100], average Shannon wavelet [101], average Rényi wavelet [101], average Tsallis wavelet [101], inherent [102], fractional fuzzy [103], and average fuzzy [104]; all of these methods will be covered and fully explained in the third section.

In the classification step, a variety of classification methods based on ML methods and DL are used. Classification techniques based on ML involve the support vector machine (SVM) [105] and k-nearest neighbors (KNN) [106] methods. The DL method is a CNN–RNN with the proposed number of layers and is another the novelty of the paper.

The proposed CNN–RNN model has two inputs. In the first input, handcrafted features will be fed into the network. In the second input, raw EEG signals of each dataset will be fed into the network differently, and various features will be extracted after passing the convolutional and long short-term memory (LSTM) layers. These features will be combined afterward and will pass into the classification algorithm.

This paper is organized as follows: the proposed method for epileptic seizure detection in EEG signals is introduced in Section 2. In Section 3, the statistical metrics for the proposed method are presented. The results of the proposed method are shown in Section 4. The limitations of the study are presented in Section 5. Finally, the discussions, conclusions, and future works are introduced in Section 6.

## 2. Materials and Methods 

### 2.1. Dataset

#### 2.1.1. Bonn Dataset

**Figure 2:**
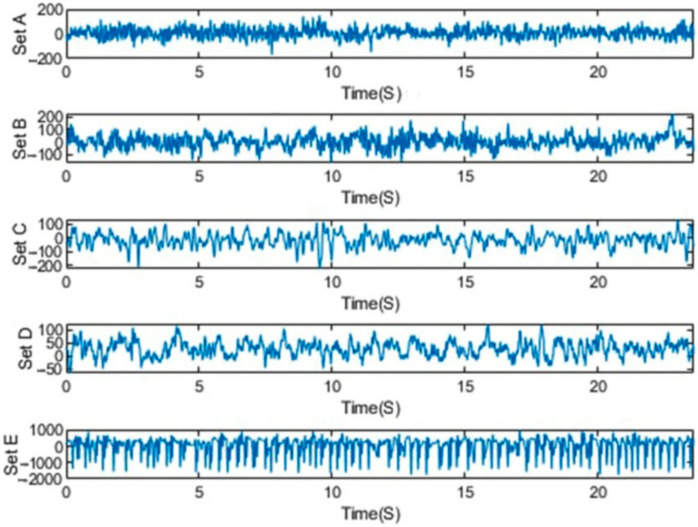
Exemplary EEGs from five datasets.

The Bonn dataset was recorded at the University of Bonn by a group of researchers, and it has been extensively used in the area of epileptic seizure analysis and detection [48]. The Bonn dataset is publicly available as 500-EEG single-channel data. It was sampled at 173.6 Hz with a 23.6 s duration. They consisted of five classes, viz., S, F, N, O, and Z, with 100 channel recordings in each class [79]. Five healthy controls in the relaxed and awake state with 10–20 standard electrode placement schemes contributed to the classes O and Z EEG surface data. Intracranial electrodes were used with five patients suffering from epilepsy to collect data of the S, F, and N classes. The hemisphere of the epileptogenic zone and the opposite hemisphere were used, respectively, for the recording of the F and S classes’ signals during the interictal (seizure-free) period. The ictal (seizure) period was taken into account in case of the recording of class S [79]. Samples of EEG signals of the dataset for each class are shown in Figure 2.

Other details about the Bonn dataset are shown in Table 1.

To perform the experiments, 6 different classification problems are used, which are shown in Table 2.

#### 2.1.2. Freiburg Dataset

The Freiburg dataset is another most frequently used resource for epileptic seizure detection [80]. It is also a freely accessible and downloadable EEG recording dataset. Twenty-one epileptic patients were considered for 24 h invasive presurgical continuous EEG signal recordings. During the time period, many seizures were recorded and occurred. This dataset includes epileptic seizure types of tonic–clonic (GTC), complex partial (CP), and simple partial (SP). Each of the cases has at least two types of epileptic seizures. The patients were from different age groups. They also differed in type and locality of seizures. The patients came to the University Hospital of Freiburg, Germany for a presurgical diagnosis. A Neurofile NT digital video EEG was used with a 256-Hz sampling rate and 128 channels [80]. The channels were numbers from 1 to 6, where the 1–3 channels were for focal recoding and 4–6 channels corresponded to extra focal ones. Interictal and ictal were the two types of signal files. The duration of the EEG signals for each patient in the ictal files was one hour. The format of the data files was ASCII. More details about this dataset is described in Table 3.

### 2.2. Preprocessing

#### Tunable-Q Wavelet Transform

The TQWT method is described in this section. TQWT is one of the newest wavelets transforms that is widely used in the processing of biological signals such as EEG signals. In TQWT, the redundancy (*r*), number of frequency sub-bands (*J*), and Q-factor (*Q*) can be tuned. The TQWT method consists of two low-pass and high-pass filter banks and is used to decompose EEG signals into different sub-bands. In this section, the low- and high-pass scale factors for filter banks with two channels are represented by α and β. The low-pass filter frequency response can be described as follows [81]:

(1) $$T0\left(\omega \right)=\left\{\begin{array}{c} 1\;\;\;\;\;\;\;\;\;\;\;\;\;\;if\;\left|\omega \right|<\left(1\;–\alpha \right)\pi \;\\ \theta \left(\frac{\omega +\left(\alpha -1\right)\pi }{\beta +\alpha -1\;}\right)\;\;\;\;\;\;if\;\left(1-\alpha \right)\pi \leq \;\left|\omega \right|<\beta \pi \\ 0\;\;\;\;\;\;\;\;\;\;\;\;\;if\;\beta \pi \leq \;\left|\omega \right|<\pi \end{array}\right.$$

The mathematical expression for the high-pass filter frequency response is as follows:

(2) $$T1\left(\omega \right)=\left\{\begin{array}{c} 0\;\;\;\;\;\;\;\;\;\;\;if\;\left|\omega \right|<\left(1\;–\alpha \right)\pi \\ \theta \left(\frac{\beta \pi -\omega }{\beta +\alpha -1\;}\right)\;\;if\;\left(1-\alpha \right)\pi \leq \;\left|\omega \right|<\beta \pi \\ 1\;\;\;\;\;\;\;\;if\;\beta \pi \leq \;\left|\omega \right|<\pi \end{array}\right.$$

**Figure 3:**
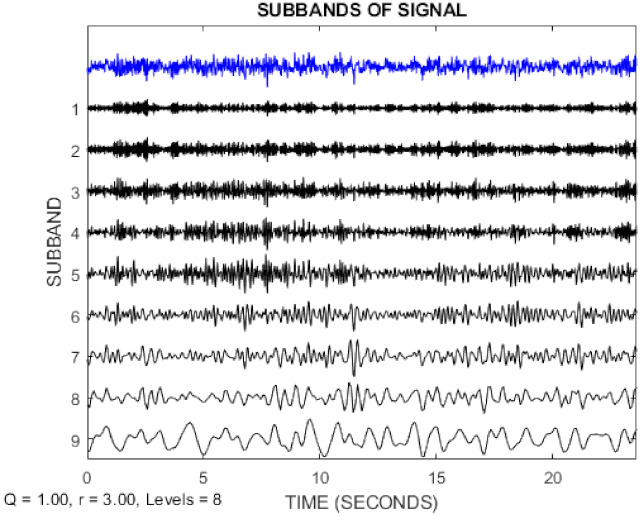
EEG signal decomposition using TQWT for the Bonn dataset.

**Figure 4:**
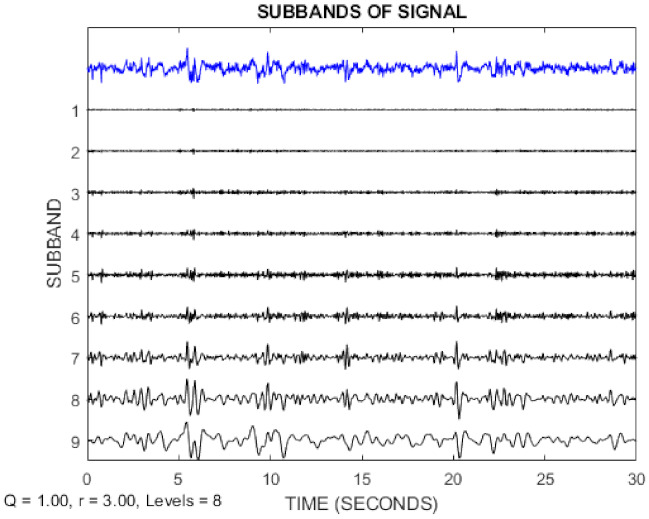
EEG signal decomposition using TQWT for the Freiburg dataset.

In this paper, the TQWT parameters for the two datasets are *r* = 3, *Q* = 1, and *J* = 8, respectively. Figure 3 and Figure 4 show the TQWT sub-bands for the Bonn and Freiburg datasets. In Figure 3 and Figure 4, EEG signals with different sub-band frequencies are shown. The selection of the EEG signal decomposition level was made similar Reference [81].

**Figure 5:**
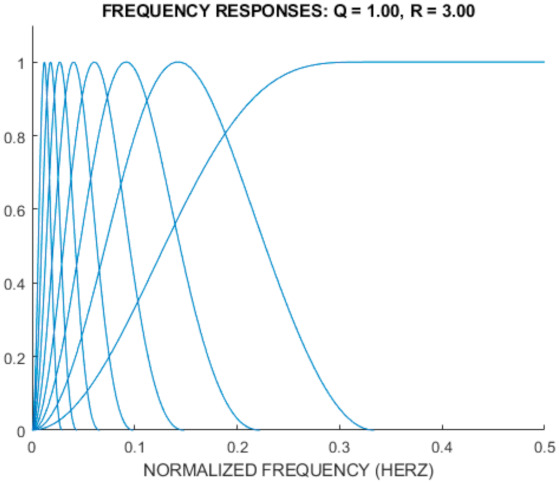
The frequency response for TQWT with the *R* = 3, *Q* = 1, and *J* = 8 parameters.

Additionally, Figure 5 shows the frequency response for TQWT based on the *r* = 3, *Q* = 1, and *J* = 8 parameters.

### 2.3. Feature Extraction

In this section, various feature extraction methods are employed in epileptic seizure detection in the EEG signals. The feature extraction methods in the EEG signals contain the statistical, frequency domain, and nonlinear features. The nonlinear features are based on fractal theory entropy techniques. In the following section, each of these methods is discussed.

#### 2.3.1. Statistical Features

The statistical features extract useful signal information, the most important of which are selected as shown in Table 4 [24].

#### 2.3.2. Frequency Features

(1) Intensity Weighted Mean Frequency (IWMF)

The intensity weighted mean frequency (IWMF) or mean frequency is an average frequency that is calculated as the sum of the product of the normalized power spectral density (PSD) and the frequency. Consider $x\left[k\right]\;$ as the normalized PSD of the signal epoch at the frequency of $\;f\left[k\right]$, and the *IWMF* is calculated by [107]

(9) $$IWMF\left(x\right)=\underset{k}{\sum }x\left[k\right]f\left[k\right]\;$$

(2) Intensity Weighted Bandwidth (IWBW)

The weighted standard deviation of the frequency and a measure of the PSD width can be obtained from [107].

(10) $$IWBW\left(x\right)=\sqrt{\underset{k}{\sum }x\left[k\right]{\left(f\left[k\right]-IMWF\left(x\right)\right)}^{2}}$$

where *x*[*k*] is the normalized PSD, and *IMWF* is the mean frequency of the input signal epoch. Whenever the PSD changes sharply, it results in a lower IWBW [107].

#### 2.3.3. Fractal Features

The fractal dimensions (FDs) are an important class of nonlinear features and play a crucial role in the processing of EEG signals. FD-based feature extraction techniques, due to their properties, increase the accuracy of epileptic seizures detection in EEG signals. In this paper, the most important FDs, including Higuchi, Katz, Petrosian, and DFA are used to epileptic seizures detection in EEG signals. In the following, each of the FDs methods is presented along with their mathematical equations.

(1) Higuchi Fractal

In this section, the theory of the Higuchi method is presented. Higuchi proposed this method in 1988, after which it has become a widely used technique for analyzing time series [88]. The Higuchi method is one of the most important FDs techniques that work well on nonlinear time series such as EEG signals. In the following, the steps of the Higuchi algorithm are proposed [88].

Consider $x\left(1\right),\;x\left(2\right),\ldots ,\;x\left(N\right)$ the time sequence to be examined. The new time series $x_{m}^{k}$ is as follows [88].

(11) $$x_{m}^{k}=\left\{x\left(m\right),x\left(m+k\right),x\left(m+2k\right),\ldots \ldots .x\left(m+\frac{N-m}{k}k\right)\right\},\;for\;m=1,2,\ldots k$$

In Equation (11), k is means the discrete time interval between points, and m is means the initial time value. For each time series $x_{m}^{k}$, the average length $L_{m}\left(k\right)$ is as follows [88].

(12) $$L_{m}\left(k\right)=\frac{\left(N-1\right)}{\lfloor \frac{N-m}{k}\rfloor k}\overset{\left\lfloor \left(N-m\right)/k\right\rfloor }{\underset{i=1}{\sum }}|x\left(m+ik\right)-x(m+\left(i-1\right)k|$$

In Equation (12), $\frac{\left(N-1\right)}{\lfloor \frac{N-m}{k}\rfloor k}$ is a normalization factor, and N is the total length of the sequence of the data x. The delay k is computed for all EEG data with an average length *k* as the mean of the k lengths $L_{m}\left(k\right)$ for m=1,2,…,k. For each *k* ranging from 1 to $k_{max}$_,_ the procedure is repeated, producing the sum of the average lengths $L\left(k\right)$ for each *k* as indicated below [88].

(13) $$L\left(k\right)=\overset{k}{\underset{m=1}{\sum }}L_{m}\left(k\right)$$

(2) Katz Fractal

The FD of a curve can be termed as [88].

(14) $$D=\frac{\log_{10}\left(L\right)}{\log_{10}\left(d\right)}\;$$

In Equation (14), d is the estimated diameter as the distance between the points of the sequence. Also, *L* parameter is the total length of the curve. The equation of the d is as follows [88]:

(15) $$d=max\left(distance\left(1,i\right)\right)$$

In Equation (15), Point *i* is the one that maximizes the first point. The measurement units used depends on the computed FDs. The FDs are different if the units are different. Katz’s approach tries to resolve the issue by creating a general unit. The average step between successive points, a normalizes the distance [88]:

(16) $$D=\frac{{log}_{10}\left(L/\underline{a}\right)}{{log}_{10}\left(d/\underline{a}\right)}$$

where *n* is the number of steps in the curve. Finally, Katz’s approach for feature extraction in EEG signals is defined as follows [88]:

(17) $$D=\frac{{log}_{10}\left(n\right)}{{log}_{10}(\frac{d}{L})+{log}_{10}\left(n\right)}\;$$

(3) Petrosian Fractal

This section presents the theory of the Petrosian method. In the Petrosian method, rapid FD estimation is performed, and the results show that this method has satisfactory results. The mathematical theory of the Petrosian method is shown in (18) [88]:

(18) $$D=\frac{{log}_{10}n}{{log}_{10}n+{log}_{10}\left(\frac{n}{n+0.4\;N∆}\right)}$$

(4) Detrended Fluctuation Analysis

The Reference [89] introduced DFA, which can be used in feature extraction from time series such as EEG signals. The RR interval of the time series is incorporated $y\left(k\right)$ and divided into nonoverlapping and equal segments of length n for conducting such an analysis. Least squares fitting is applied to obtain the local trend $y_{n}\left(k\right)$ in each segment and subtracted from $y\left(k\right)$. $F\left(n\right)$, the root mean square fluctuation estimates, are calculated at last, and the scaling exponents are measured as the slope of the double-log plot of *F(n)* against *n* [89,90]:

(19) $$F\left(n\right)=\sqrt{\frac{1}{N}\overset{N}{\underset{k=1}{\sum }}{\left[y\left(k\right)-y_{n}\left(k\right)\right]}^{2}}$$

#### 2.3.4. Entropy Features

In this paper, different entropies are exploited to extract the characteristics of EEG signals. The entropy-based features indicate the presence of signal irregularities and are also more resistant to noise than other methods. The entropy relationships used are shown below.

(1) Shannon Feature

This entropy was proposed by Reference [94] and defined as

(20) $$E_{Sh}=-\overset{x}{\underset{n=1}{\sum }}S_{n}{log}_{2}S_{n}$$

In Equation (20), *S_n_* is the probability of the feature’s value.

(2) Log-Energy Entropy

The log-energy entropy estimates the complex intensity of the signals. The log-energy entropy can be termed as [91,93]

(21) $$E_{Log}=\overset{K}{\underset{i=0}{\sum }}log\left(E_{i}^{2}\right)$$

(3) Average Shannon Wavelet Entropy

In this section, the average entropy of wavelet Shannon is presented. If $E_{t}$ represents the energy of the 1st sub-band signal calculated from the wavelet coefficients, we can write the total energy of the signal as follows [101]:

(22) $$E_{t}=\overset{K}{\underset{i=1}{\sum }}E_{i}$$

where *K* represents the total number of EEG signals obtained from the wavelet sub-bands. The wavelet energy can be calculated as follows [101]:

(23) $$q_{i}=\frac{E_{i}}{E_{t}}$$

The Shannon-based wavelet entropy relationship is defined as follows [101]:

(24) $$Swn=-\overset{K}{\underset{i=1}{\sum }}q_{i}log(q_{i})$$

Finally, the average wavelet Shannon entropy is defined based on $swn_{x}$ and $swn_{y}$, which represent the Swn of the time series *x* and *y* of the EEG signal, as follows [101]:

(25) $$swn_{avg}=\frac{swn_{x}+swn_{y}}{2}$$

(4) Average Rényi Wavelet Entropy

The entropy of wavelet Rényi is defined in Relation (26) [101]:

(26) $$Rwn_{\alpha }=\frac{1}{1-\alpha }log\left(\overset{K}{\underset{i=1}{\sum }}q_{i}^{\alpha }\right),\;\alpha \neq 1\;$$

Here, the parameter *α* is considered equal to 2. In another definition, Rényi entropy is expressed by Relation (27) [101]:

(27) $$Rwn_{2}=-log\left(\overset{K}{\underset{i=1}{\sum }}q_{i}^{2}\right)$$

Similar to Equation (25), the average wavelet Rényi entropy is defined as follows [35]:

(28) $$Rwn_{Avg}=\frac{Rwn_{x}+Rwn_{y}}{2}$$

(5) Average Tsallis Wavelet Entropy

In Reference [101], the entropy of wavelet Tsallis is studied in detail. Wavelet Tsallis entropy is defined as follows:

(29) $$Twn_{\alpha }=\frac{1}{1-\alpha }\left(1-\overset{K}{\underset{i=1}{\sum }}q_{i}^{\alpha }\right),\;\alpha \neq 1\;$$

where parameter a is called the nonextensivity index. The average wavelet Tsallis entropy is calculated as follows [101]:

(30) $$Twn_{Avg}=\frac{Twn_{x}+Twn_{y}}{2}\;$$

(6) Permutation Rényi Entropy

Consider the following time series. The $X_{t}$ vectors are constructed by selecting samples with identical distances from *x*, starting from the time point *t* [100]:

(31) $$X_{t}=[x\left(t\right),x\left(t+L\right),\ldots ,x{\left(t+\left(m-1\right)L\right]}^{T}$$

The values of $X_{t}$ are transformed in ascending order and, by generating $X_{r_{t}}$, the modified version of $X_{t}$, the time points are renamed [100]:

(32) $$X_{r_{t}}={\left[x\left(t+\left(t_{1}-1\right)L\right),\;x\left(t+\left(t_{2}-1\right)L\right),\ldots ,x\left(t+\left(t_{m}-1\right)L\right)\right]}^{T}$$

Therefore, each $X_{t}$ vector can be considered uniquely mapped on a symbol vector *π* = [*t*_1_, *t*_2_,…, *t_m_*]. PE can be calculated as follows [100]:

(33) $$H\left(m\right)=-\overset{m!}{\underset{i=1}{\sum }}p\left(\pi_{i}\right)log\left(p\left(\pi_{i}\right)\right)$$

where *log* is a natural logarithm, and m! is the number of possible permutations. Since $H\left(m\right)$ can reach *ln* (m!), PE is normalized. Then, the normalized PE relationship is defined by [100].

(34) $$H_{n}\left(m\right)=-\frac{\sum_{i=1}^{m!}p\left(\pi_{i}\right)log\left(p\left(\pi_{i}\right)\right)\;}{ln\left(m!\right)}$$

Here is a new definition of PE based on Rényi’s theory as follows [100]:

(35) $$H_{R}\left(m\right)=-\frac{1}{1-\alpha }log\overset{m!}{\underset{i=1}{\sum }}p{\left(\pi_{i}\right)}^{\alpha }\;$$

(7) Graph Entropy

A new entropy method based on graph theory was proposed by Reference [99]. The relation of the graph entropy is described as [99].

(36) $$H_{n}\left(m\right)=-\frac{\sum_{i=1}^{m!}p\left(\pi_{i}\right)log\left(p\left(\pi_{i}\right)\right)\;}{ln\left(m!\right)}$$

(37) $$H_{n}\left(m\right)=-\frac{\sum_{i=1}^{m!}p\left(\pi_{i}\right)log\left(p\left(\pi_{i}\right)\right)\;}{ln\left(m!\right)}$$

where $W_{ij}$ is the weight of the link between the *i*th node and the *j*th node, and m is the number of nodes connected to the *i*th node [99].

(8) Fuzzy Entropy

For a time series $x\left(i\right)$, fuzzy entropy (FuEn) establishes vector sequences $x_{i}^{m},\;i=\left\{1,2,\ldots ,N-m+1\right\}$ as given below [97]:

(38) $$X_{i}^{m}=\left\{x\left(i\right),x\left(i+1\right),\ldots ,x\left(i+m-1\right)\right\}-x_{0}\left(i\right)$$

where *m* is the length of the sequences.

$D_{ij}^{m}$ is the maximum absolute difference between $X_{i}^{m}$ and $\;X_{j}^{m}$ [97].

(39) $$D_{ij}^{m}\left(n,r\right)=\mu \left(d_{ij}^{m},n,r\right)$$

(40) $$\mu \left(d_{ij}^{m},n,r\right)=e^{-\frac{{\left(d_{ij}^{m}\right)}^{n}}{r}}$$

In Equations (40) and (41), r parameter is the predefined gradient, and n is the width of the exponential function. The $\Phi^{m}$ function shows in the Eqation (41) [97]:

(41) $$\Phi^{m}\left(n,r\right)=\frac{1}{N-m}\overset{N-m}{\underset{i=1}{\sum }}\left(\frac{1}{N-m-1}\overset{N-m}{\underset{j=1,j\neq i}{\sum }}D_{ij}^{m}\right)$$

Finally, the FuEn is introduced as Equation (42) [97]:

(42) $$FuEn\left(m,n,r,N\right)=-ln\frac{\Phi^{m+1}\left(r\right)}{\Phi^{m}\left(r\right)}$$

(9) Refined Composite Multiscale Fuzzy Entropy (RCMFE)

The RCMFEσ is computed as follows [98]:

(43) RCMFEσ (x, m, n, r)=−lnΦ¯rm+1Φ¯rm

RCMFEσ and RCMFEμ have differences that both use different equations in the first steps of their algorithms. The tolerance (*r*), Fuzzy entropy power (*n*), and the embedding dimension (*m*) [98].

(10) Inherent Fuzzy Entropy

This section expresses inherent fuzzy entropy (IFuEn). The steps of IFuEn are as follows [102]:

Step 1. Multiple IMFs are made by breaking down the original *x*(*t*) signal and reconstructing the $\hat{x}\left(t\right)$ signal using EMD techniques, which are done as follows [102]:

1. Calculating the extremes to cover $e_{min}\left(t\right)$ and $e_{max}\left(t\right)$ [102].
2. Calculating the average [102]:
  (44) $$m\left(t\right)=\left(\frac{e_{min}\left(t\right)+e_{max}\left(t\right)}{2}\right)$$
3. Candidates of inherent functions are derived intrinsic mode functions (IMFs) [102]:
  (45) $$d\left(t\right)=x\left(t\right)-m\left(t\right)$$
4. Calculating the value of *r*(*t*) as follows [102]:
  (46) $$r\left(t\right)=x\left(t\right)-\overset{t}{\underset{i=1}{\sum }}d\left(t\right)$$
5. Given *t* = *t* + 1, consider *d(t +* 1) as the input EEG data; while iterating on the residual *m*(*t*), which continues until the final residue *r* that becomes a monotonic function from which no more IMF can be extracted [102].
6. The total accumulated residual IMFs are used to reconstruct the $\hat{x}\left(t\right)$ signal [102]:
  (47) $$\hat{x}\left(t\right)=\overset{i=m}{\underset{i=n}{\sum }}d\left(t\right)$$

Step 2: *FuEn* to evaluate the complexity, which is similar to Equation (42) [102].

Step 3: Multi-scale version [102]

$y_{j}^{\left(\tau \right)}$ is the coarse-grained time series, and its equation is as follows [102]:

(48) $$y_{j}^{\left(\tau \right)}=\frac{1}{\tau \sum_{i=\left(j-1\right)\tau +1}^{j\tau }x_{i}}$$

In this regard, *τ* is the scale factor. Also, the length of each coarse-grained time series is *N*/*τ* [102].

(11) Averaged Fuzzy Entropy

Average fuzzy entropy (AFuEn) is an improved model of FuEn. In AFuEn method, an improved m_ pattern $\Gamma_{k}\left[X_{j}^{m}\right]$ is compared to $X_{i}^{m}$. At this AFuEn, Equation (49) is modified as follows [104]:

(49) D kijmn,r=μdXim,ΓkXjm,n,r

In the following, four different types of $\Gamma_{k}\left[X_{m}\left(j\right)\right]\;$ operations with $k=\left\{T,R,I,G\right\}$ are defined as follows [104]:

- A translation of *n* samples, k=T corresponds to $\Gamma_{T}\left[X_{j}^{m}\right]=X_{j+n}^{m}$.
- A reflection at the position *n*, k=R corresponds to $\Gamma_{R}\left[X_{j}^{m}\right]=X_{-j+n}^{m}$.
- An inversion at the position *n*, k=I corresponds to $\Gamma_{I}\left[X_{j}^{m}\right]=-X_{-j+n}^{m}$.
- A glide reflection of *n* samples, k=G corresponds to $\Gamma_{G}\left[X_{j}^{m}\right]=-X_{j+n}^{m}$.

In this case, $FuEn_{T}$, $FuEn_{R}$, $FuEn_{I}$, and $FuEn_{G}$ are obtained. The following $FuEn_{a}$ is as follows [104]:

(50) $$FuEn_{a}\left(m,n,r,N\right)=\frac{\left(FuEn_{T}+FuEn_{R}+FuEn_{I}+FuEn_{G}\right)}{4}\;$$

Finally, the AFuEn is shows as Equation (51) [104]:

(51) $$AFuEn\left(m,n,r,N\right)=ln\left(\frac{\Phi_{k}^{m}\left(n,r\right)}{\Phi_{k}^{m+1}\left(n,r\right)}\right)$$

(12) Fractional Fuzzy Entropy

In Reference [103], researchers introduced the fractional-order entropy of Shannon, which is defined as

(52) $$S_{\alpha }=\underset{i}{\sum }p_{i}\left\{-\frac{p_{i}^{-\alpha }}{\Gamma \left(\alpha +1\right)}\left[lnp_{i}+\psi \left(1\right)-\psi \left(1-\alpha \right)\right]\right\}\;$$

In Equation (52), *α* is the fractional-order derivation. Moreover, Γ and ψ denote the gamma and digamma functions, respectively. The equation of fractional-order information is defined as Equation (53):

(53) $$I_{\alpha }=-\frac{p_{i}^{-\alpha }}{\Gamma \left(\alpha +1\right)}\left[lnp_{i}+\psi \left(1\right)-\psi \left(1-\alpha \right)\right]\;$$

In Equation (42), *FuEn* is introduced. Placing Equation (53) in Equation (42), fractional fuzzy entropy (*FFuEn*) may be stated as

(54) $$FFuEn\left(m,r,\alpha ,x^{N}\right)=-{\left(\frac{\Phi^{m+1}\left(r\right)}{\Phi^{m}\left(r\right)}\right)}^{-\alpha }\frac{ln\frac{\Phi^{m+1}\left(r\right)}{\Phi^{m}\left(r\right)}+\psi \left(1\right)-\psi \left(1-\alpha \right)}{\Gamma \;\left(1+\alpha \right)}\;$$

(13) Spectral Entropy

This method is normalized Shannon entropy, which quantitatively defines the spectral complexity of the EEG signals as follows [94]:

(55) $$S_{ent}=\underset{f}{\sum }P_{f}log\left(\frac{1}{P_{f}}\right)$$

(14) Sample Entropy

In the equation below, the sample entropy formula is shown [95]:

(56) $$SampEn=-log\left(\frac{A}{B}\right)$$

where *A* refers to the total number of vector pairs of length *m* + 1, and *B* comprises the total number of vector pairs of length *m* [95].

(15) Permutation Entropy

Permutation entropy estimates the complexity of biomedical signals, such as EEG signals, by measuring the couplings between two classes. The equation of permutation entropy is presented as follows [96]:

(57) $$PE=-\overset{n}{\underset{j=1}{\sum }}P_{j}log_{2}P_{j}$$

where *n* defines the sequence length, and $p_{j}$ illustrates the likelihood of the *n*th occurrence [96].

### 2.4. Classification

#### 2.4.1. SVM

While these methods have been around for longer than many other machine learning algorithms, in recent decades, despite many advances in machine learning and the introduction of a wide variety of novel algorithms, support vector machines [105] have not lost their popularity and are still considered one of the most well-known and applied methods among researchers. These algorithms, which are generally based on finding hyperplanes that maximize the margin, use the kernel trick to classify data in complex and high-dimensional spaces with suitable accuracy. Linear, RBF, and polynomial are the most popular SVM kernels [105].

#### 2.4.2. KNN

One of the simplest and, at the same time, most practical machine learning methods is the KNN algorithm [106], which is widely used for classification. There is no learning phase in this method, but in the test phase, the classifier finds the K-nearest neighbor to this data point (as the name of the method implies) and assigns the data label according to their dominant label. Nevertheless, this method works very slowly in times when the amount of training data is enormous [106].

#### 2.4.3. CNN–RNN

In this section, the proposed DL architecture for the detection of epileptic seizures based on EEG signals is discussed. The network used in this paper has a CNN–RNN structure with the use of extracted features. Nowadays, combined deep learning models such as CNN–RNN have achieved successful results in diagnosing and predicting diseases from medical data.

Convolutional layers are usually used in the primary layers to combine CNN models with RNN, which are responsible for extracting the features. The output of the convolutional layers is then applied to the RNN layers to use their superiority to identify the global pattern [108,109]. The purpose of this work is because the convolution layers empirically find local and spatial patterns far better than RNNs in signals [109]. Second, adding convolution layers allows the RNN to see the data faster, thus finding more distance patterns. Additionally, in this study, it has been proven that combining handcrafted features with CNN–RNN networks helps to increase the efficiency and accuracy of the CADS detection of epileptic seizures from EEG signals. In this study, the selection of the number of layers of the CNN–RNN model is presented for the first time by the researchers in this paper.

**Figure 6:**
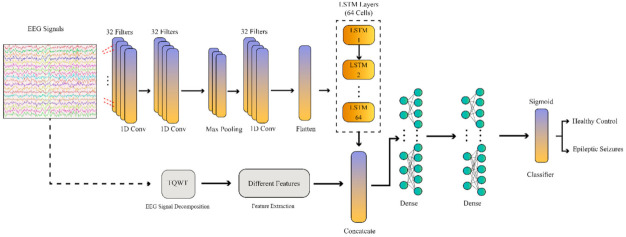
Block diagram of the proposed CNN–RNN network.

In this paper, a deep CNN–RNN network with the proposed number of layers, along with handcrafted features, is used to diagnose epileptic seizures. The proposed CNN–RNN model is applied to the Bonn and Freiburg datasets, along with the handcrafted features. The CNN–RNN model has the same structure for both datasets. Figure 6 shows the proposed CNN–RNN model. Additionally, the hyper parameters of the model are shown in Table 5.

In the proposed deep learning method, there are three convolutional layers in the convolutional section to extract features and one max-pooling layer with a feature reduction purpose. After that, there is a flatten layer to transform the extracted features into feature vectors. Then, an LSTM block with 64 neurons was used to extract the RNN features. Afterwards, a combination block was used to combine the CNN–RNN and handcrafted features. Finally, three fully connected layers were implemented to classify the data. In the proposed CNN–RNN model, each layer’s selection and its parameters were made by trial and error.

## 3. Statistical Metrics

In this paper, the classification results are evaluated using the 10-fold cross-validation techniques. In K-fold cross-validation, the total number of observations are split into K-folds, where the data samples are limited. Finally, the performance of the algorithm was estimated using statistical metrics include specificity (*Spec*), sensitivity (*Sens*), accuracy (*Acc*), and F1-score (F1-S), and precision (*Prec*). The true positive (*TP*), true negative (*TN*), false negative (*FN*), and false positive (*FP*) parameters are extracted from the confusion matrix [110].

(58) $$Acc=\frac{TP+TN}{FP+FN+TP+TN}$$

(59) $$Sens=\frac{TP}{FN+TP}$$

(60) $$Spec=\frac{TN}{FP+TN}$$

(61) $$Prec=\frac{TP}{TP+FP}$$

(62) $$FS=\frac{2\;TP}{2TP+FP+FN}$$

## 4. Results

The experiments are performed on a Ryzen 1700 machine with 8-GB RAM using MATLAB for feature extraction and TensorFlow 2 and scikit-learn for the classification algorithms. In this part of the paper, we present the results of the proposed method. The proposed method includes the preprocessing, feature extraction, and classification steps. The preprocessing step includes windowing, noise removal, and decomposition of the EEG signals into various sub-bands by the TQWT. In the first step of preprocessing, the signals from the Bonn and Freiburg datasets are decomposed into different time windows. For the Bonn dataset, each EEG signal is segmented into time windows of 5 s, and for the Freiburg dataset, each EEG signal is segmented into time windows of 4 s. In the following, a Butterworth band-pass filter is used to preprocess the signals of the datasets. In the third preprocessing step, TQWT is used for EEG signal decomposition. As mentioned earlier, the important TQWT parameters are selected as *Q* = 1, *r* = 3, and *J* = 8 for both datasets.

In the following, various statistical, frequency, and nonlinear features are extracted from the TQWT sub-bands. The combination of these features has been done for the first time in this paper and is considered an important novelty.

In the final part of CADS, the epileptic seizure detection based on EEG signals, ML classifier algorithms, and deep learning was examined and tested. The ML classifier techniques include SVM and KNN methods. On the other hand, the DL method is a CNN–RNN model. This method of classification is another novelty of this paper. Here, the proposed CNN–RNN method has two separate inputs. In the first input, Bonn or Freiburg dataset signals are applied to one of the proposed CNN–RNN network inputs. After passing the raw signals of the datasets through the one-dimensional (1D) convolutional layers, they finally reach the flatten layer. On the other hand, handcrafted feature extraction methods are applied to the second input of the proposed CNN–RNN architecture (Figure 6). Then, the handcrafted features and the features extracted from the 1D convolutional layers are merged and passed through the RNN layers to be finally classified.

In the proposed CNN–RNN implementation on the Bonn dataset, each data is broken into 5 s windows, and after preprocessing, some features are extracted from it. At the same time, each 5 s window, which contains 868 frames of data, is broken by 25 overlaps into 33 windows, each containing 50 frames, which are used for CNN–RNN input as the raw data. In the proposed method, each 100-epoch network is trained using the categorical cross-entropy error function and Adam optimizer.

It is also important to note that the implementation and configuration of the proposed CNN–RNN model for the Freiburg dataset are similar to the Bonn dataset. As can be seen in Table 6 and Table 7, the proposed CNN–RNN model has been successful in epileptic seizure detection from the Bonn and Freiburg datasets. In Table 6, the different classifications are reviewed.

**Figure 7:**
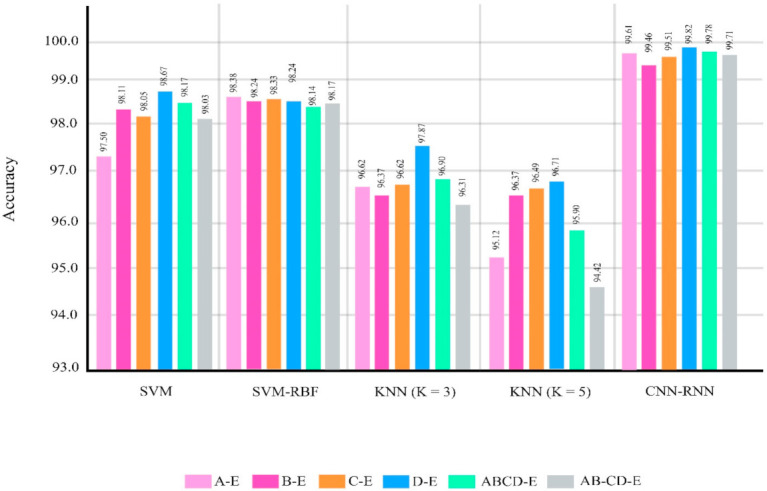
Results for different methods in different classification problems of the Bonn dataset.

The classifications were chosen similar to the research papers about epileptic seizure diagnosis based on EEG signals using the Bonn dataset [21,22,23,24,25,26,27,28,29,30,31,32,33,34,35,36,37,38,39,40,41,42,43,44,45,46,47,48,49,50,51,52,53,54,55,56,57,58,59,60,61,62]. Figure 7 and Table 6 show the performances of different classifier methods for the Bonn dataset.

Additionally, the results of the Fribourg dataset are shown in Table 7.

## 5. Limitations of Study

In this section, the limitations of the study are discussed. As mentioned before, epileptic seizures have various types, and their on-time diagnosis has great importance. There has been no dataset on the types of epileptic seizures so far. Therefore, researchers cannot do serious research in this field. In addition, the available EEG datasets for epileptic seizure diagnosis have limited use, and achieving actual and accurate epileptic seizure detection based on AI techniques is not possible due to this limitation. Another limitation of epileptic seizure diagnosis from EEG signals is that there are no dataset of EEG signals with preictal, ictal, and interictal times being highlighted in them. In the case of addressing these limitations, it is possible to use advanced and novel DL models to diagnose various types of epileptic seizures.

## 6. Discussion, Conclusions, and Future Works

Epileptic seizures are defined as a group of neurological disorders, and their early diagnosis is of particular importance for specialist physicians and neurologists [82,111]. In order to epileptic seizures detection, several techniques have been proposed until now. Among the neuroimaging modalities, EEG is pivotally significant to specialist physicians compared to other modalities. EEG signals provide specialist physicians with accurate information about brain functions, which helps to accurately diagnose epileptic seizures. EEG signals, though very beneficial, are not bereft of disadvantages and always cause problems for specialist physicians. Long-term recording, multiple EEG channels, various noises in EEG signals, etc. are some of the physicians’ difficulties that pose problems for accurately and quickly diagnosing epileptic seizures.

So far, various AI methods have been proposed to epileptic seizures detection, aiming to aid specialist physicians in the rapid diagnosis of epileptic seizures based on EEG signals. Researchers in the past have mostly exploited ML methods to diagnose epileptic seizures. Inefficiency in large amounts of input data, the complexity of the methods, the need for great knowledge to use ML methods in diagnosing epileptic seizures, etc. are the most important deficiencies of these methods. To address this issue, in recent years, DL approaches have been proposed that possess appropriate efficiency and performance for diagnosing various diseases, including epileptic seizures, by using a large amount of input data.

The proposed method consisted of three parts: preprocessing, feature extraction, and classification. Two datasets, Bonn and Freiburg, were exploited for the experiments. Bonn dataset signals were selected for 5 s time windows and Freiburg dataset signals for 4 s time windows. In the preprocessing step, first, a Butterworth band-pass filter was utilized for the initial preprocessing of the two dataset signals. Following the preprocessing step, the TQWT technique was adopted to decompose the EEG signal datasets into different su-bands. The TQWT parameters were selected to be applied to the two datasets similar to Reference [82].

In the following, a variety of statistical, frequency, and nonlinear features were extracted from TQWT sub-bands. Statistical features contain statistical moments. Nonlinear features also involve two categories of FDs and entropies. FD-based nonlinear features include Higuchi, Katz, Petrosian, and DFA. Entropy-based feature extraction techniques also include Shannon, Log-Energy, spectral, Sample, permutation, Fuzzy, refined composite multiscale fuzzy, graph, Permutation Rényi, average Shannon wavelet, average Rényi wavelet, average Tsallis wavelet, inherent, fractional fuzzy, and average fuzzy. In the feature extraction section, for the first time, a combination of these features has been used to epileptic seizures detection based on EEG signals and is considered the first novelty of this article.

Finally, ML methods and a CNN–RNN based on a DL model were exploited in the classification step. Among the classification methods, the CNN–RNN was applied for the first time in this study and was carried to account for another novelty. Here, the proposed CNN–RNN approach entailed two separate inputs. In the first input, the EEG signals of the Bonn or Freiburg datasets were fed to one of the proposed CNN–RNN network inputs. After the raw signals of the datasets passing through the 1D convolutional layers, they eventually attained the flatten layer. On the other hand, handcrafted feature extraction methods were applied to the second input of the proposed CNN–RNN architecture (Figure 6). Then, the handcrafted features and the features extracted from the one-dimensional convolutional layers were combined and passed through the RNN layers to finally be classified. In the classification section, K-fold cross-validation with K = 10 was used to calculate the valid outcomes. The proposed CNN–RNN architecture is a novel feature fusion procedure. Among the advantages of the proposed architecture, its high accuracy and greater efficiency in practical applications can be meaningful. The results identified that the proposed CNN–RNN scheme was able to achieve the maximum level of accuracy among all the algorithms used.

Then, in Table 8 and Table 9, the researchers conducted on the Bonn and Freiburg datasets for the diagnosis of epileptic seizures using AI methods are presented and compared with the proposed method.

According to Table 8 and Table 9, it can be perceived that the proposed CADS for the diagnosis of epileptic seizures using the handcrafted features and the proposed CNN–RNN model have achieved successful results.

As shown in Table 8 and Table 9, the proposed method could improve the performance and accuracy of an epileptic seizure diagnosis in the Bonn and Freiburg datasets. The proposed method has higher performance in comparison with other research projects. Table 8 and Table 9 shows that the results are reliable, and it is possible to use this proposed method in clinical applications to diagnose epileptic seizures. The proposed method in this paper has high efficacy in the diagnosis of epileptic seizures. In this method, different handcrafted features are used in combination with DL that improved the accuracy of diagnosing epileptic seizures based on EEG signals. The proposed method can help specialists rapidly diagnose epileptic seizures. This study shows that the proposed method can be implemented on a software platform and used in hospitals.

In future works, graph theory methods will be utilized, coupled with novel handcrafted features [112,113]. Additionally, applying new fuzzy entropies as feature extraction methods can be a future work. Additionally, another future work is to use fuzzy methods [114,115] in epileptic seizure detection. In other future works, effective connectivity techniques may be used to diagnose epileptic seizures [116,117,118]; first, EEG signals are transformed into 2D images using effective connectivity methods. Then, these 2 D images are applied to different 2D deep learning networks. Another future work is using novel DL techniques such as attention learning [119,120,121,122], transformers [123,124], and other advanced deep learning techniques [125,126,127,128,129,130,131,132,133,134] for epileptic seizure detection. Finally, adopting novel deep feature fusion techniques to epileptic seizures detection based on EEG signals can be noteworthy as one of the future works [135].

## Figures and Tables

**Table 1:** A thorough explanation of the five subsets of the dataset.

Sets	Subjects				
	Patient Stage	Electrode Type	Num. of Cases	Num. of Data	Length of Segments
Set A	Eye Open	Surface	5	100	4097
Set B	Eye Close	Surface	5	100	4097
Set C	Seizure Free	Intracranial	5	100	4097
Set D	Seizure Free	Intracranial	5	100	4097
Set E	Seizure Activity	Intracranial	5	100	4097

**Table 2:** More details about the six problem classifications.

Subjects	Problem Classifications	Description
Subject 1	A–E	Healthy—Ictal
Subject 2	B–E	Healthy—Ictal
Subject 3	C–E	Interictal—Ictal
Subject 4	D and E	Interictal—Ictal
Subject 5	ABCD and E	Normal—Seizure
Subject 6	AB and CD and E	Healthy—Interictal—Seizure

**Table 3:** More details about the Fribourg dataset.

Patient	Age	Gender	Seizure Origin	Seizure Type	Number of Seizures
1	15	Female	Temporal	SP, CP	4
2	38	Male	Frontal	SP, CP, GTC	3
3	14	Male	Temporal	SP, CP	5
4	26	Female	Temporal	SP, CP, GTC	5
5	16	Female	Frontal	SP, CP, GTC	5
6	31	Female	Temporal	CP, GTC	3
7	42	Female	Temporal	SP, CP, GTC	3
8	32	Female	Temporal	SP, CP	2
9	44	Male	Frontal	CP, GTC	5
10	47	Male	Frontal	SP, CP, GTC	5
11	10	Female	Frontal	SP, CP, GTC	4
12	42	Female	Frontal	SP, CP, GTC	4
13	22	Female	Temporal	SP, CP, GTC	2
14	41	Female	Temporal	CP, GTC	4
15	31	Male	Frontal	SP, CP, GTC	4
16	50	Female	Temporal	SP, CP, GTC	5
17	28	Male	Temporal	SP, CP, GTC	5
18	25	Female	Temporal	SP, CP	5
19	28	Female	Frontal	SP, CP, GTC	4
20	33	Male	Temporal	SP, CP, GTC	5
21	13	Male	Temporal	SP, CP	5

**Table 4:** Statistic features for epileptic seizure detection.

Formula	Feature Name	Equations
$X_{mean}=\frac{1}{n}\overset{n}{\underset{1}{\sum }}x_{i}$	Mean	(3)
$X_{var}=\overset{N}{\underset{n=1}{\sum }}\left(x_{n}-AM\right)\frac{2}{N-1}$	Variance	(4)
$X_{ku}=\overset{N}{\underset{n=1}{\sum }}\left(x_{n}-AM\right)\frac{4}{\left(N-1\right)SD^{4}}$	Kurtosis	(5)
$X_{Ske}=\overset{N}{\underset{n=1}{\sum }}\left(x_{n}-AM\right)\frac{3}{\left(N-1\right)SD^{3}}$	Skewness	(6)
$X_{std}=\sqrt{\overset{N}{\underset{n=1}{\sum }}\left(x_{n}-AM\right)\;\frac{2}{n-1}}$	Standard Deviation	(7)
$Max\left(x_{n}\right)$	Max	(8)

**Table 5:** CNN–RNN hyper-parameters.

Parameters	Layer
Kernel size = 3, activation = ‘relu’, filters = 32	Conv1d
Kernel size = 3, activation = ‘relu’, filters = 32	Conv1d_1
Pool_size = 2	Maxpooling1d
Kernel size = 3, activation = ‘relu’, filters = 32	Conv1d_2
---	Flatten
Number of neurons = 64	LSTM
Number of neurons = 128, activation = ‘relu’	Dense
Number of neurons = 128, activation = ‘relu’	Dense_1
Number of neurons = 2 or 3, activation = ‘softmax’	Dense_2

**Table 6:** Results for the Bonn dataset.

Methods	Sets	Accuracy	Precision	Spec	Sens	F1-Score
Standard SVM	A–E	97.50	97.31	97.29	97.36	97.66
	B–E	98.11	98.06	98.04	98.82	98.03
	C–E	98.05	98.54	98.56	98.47	97.95
	D and E	98.67	99.11	98.43	98.62	98.48
	ABCD and E	98.17	99.03	98.18	97.26	98.26
	AB and CD and E	98.03	98.71	98.72	98.17	98.01
SVM-RBF	A–E	98.38	98.61	98.94	98.99	98.53
	B–E	98.24	99.09	98.71	99.02	98.96
	C–E	98.33	98.98	98.76	99.13	98.83
	D and E	98.24	99.86	98.83	99.22	99.03
	ABCD and E	98.14	99.17	98.31	98.72	98.97
	AB and CD and E	98.17	99.03	99.03	98.66	98.69
KNN (K = 3)	A–E	96.62	96.32	96.50	94.75	94.51
	B–E	96.37	96.24	96.23	96.49	96.37
	C–E	96.62	95.37	95.28	98.08	96.67
	D and E	97.87	98.12	98.41	98.46	98.57
	ABCD and E	96.90	94.62	96.87	95.19	94.34
	AB and CD and E	96.31	95.18	97.30	97.44	96.11
KNN (K = 5)	A–E	95.12	95.75	92.34	92.25	94.92
	B–E	96.37	96.25	98.25	98.49	97.37
	C–E	96.49	94.92	94.62	97.21	96.56
	D and E	96.71	97.77	97.72	96.73	97.75
	ABCD and E	95.90	93.21	96.38	93.50	92.34
	AB and CD and E	94.42	94.38	96.15	95.33	96.97
CNN–RNN	A–E	99.61	99.78	99.81	99.43	99.69
	B–E	99.46	99.51	99.17	99.22	99.46
	C–E	99.51	99.42	99.31	99.43	99.28
	D and E	99.82	99.59	99.68	99.82	99.61
	ABCD and E	99.78	98.71	98.91	98.83	98.81
	AB and CD and E	99.71	99.68	99.79	99.61	99.73

**Table 7:** Results for the Fribourg dataset.

Methods	Accuracy	Sensitivity	Specificity	Precision	F1-Score
SVM	97.13	97.24	97.31	97.39	97.28
SVM–RBF	97.41	97.86	97.73	97.43	97.59
3NN	96.66	96.19	95.93	96.39	97.11
5NN	96.71	96.02	96.93	96.03	96.97
CNN–RNN	99.13	98.96	98.96	99.01	99.11

**Table 8:** Comparison of the proposed method with other related works for the Bonn dataset.

Work	Preprocessing	Feature Extraction	Feature Selection	Classifiers	Accuracy
[21]	TQWT	CCEnt	PCA	LS-SVM	97.02%
[22]	TQWT	Hybrid Features	Firefly	RF	97%
[23]	TQWT	AVP, STD	No	K-NN	98.80%
[24]	TQWT	Statistic Features	No	K-NN	100%
[25]	TQWT	KNN Entropy	Wrapper	SVM	100%
[26]	TQWT	CTM, 2D-RPS plots	N/A	NA	N/A
[27]	TQWT	MvFE	No	LS-SVM	84.67%
[28]	EMD–TQWT	IP	Different Methods	LS-SVM	99%
[29]	TQWT	SC, SS, SF, SSl	No	bootstrap	100%
[30]	TQWT	Correntropies	N/A	RF	92.78%
[31]	TQWT	KnnEnt, CCorrEnt, FzEnt	No	LS-SVM	95%
[32]	TQWT	Centered correntropy	No	RF	98.30%
[33]	TQWTRF	FDs, AppEnt	No	SVMRF	100%
[34]	TQWT	Mixture Correntropy	Various Methods	LS-SVM	90.10%
[35]	IEVDHM–HT	Various Features	Student’s *t*-test	LS-SVM	100%
[36]	FAWT	CVDistEnt, logarithmic energy	N/A	FKNN	100%
[37] Multi-Classes = 99.46%	VMD, HT	BLIMFs	No	EMRVFLN	Two-Classes = 100% Multi-Classes = 99.46%
[38] Multi-Classes = 96.50%	Filtering	LSP	NCA	SVM	Two-Classes = 99.10%
[39] Multi-Classes = 99.70%	Filtering, DWT	Different Features	N/A	SVM	Two-Classes = 99.50% Multi-Classes = 99.70%
[40]	DWT	Linear and Non-Linear Features	No	SVM	99.50%
[41]	DWT	Statistic Features, Entropy, RWE	WOA	SVM	99.80%
[42]	SSA	1D-LBP	No	SVM	N/A
[43]	DWT	Entropy Features	ANOVA-FSFS	SVM	99.50%
[44] Multi-Classes = 99.07%	WPT	FDE	Kruskal Wallis	KNN	Two-Classes = 99.69% Multi-Classes = 99.07%
[45]	MODWPT	Statistic Parameters	Different Methods	LS-SVM	99.60%
[46]	FSST	GLCM	N/A	KNN	99.59%
[47]	ECT	Graph Theory, FD	No	RF	98.50%
[48]	MRBF–MPSO	PSD	PCA	SVM	98.73%
[49]	Z-Score Normalization	1D-CNN	No	Softmax	86.67%
[50]	DWT	PSR	SVCM	LS-SVM	98.55%
[51]	EMD	Spectral and Temporal Features	No	SVM	N/A
[52]	ATFFWT	FD	Different Methods	LS-SVM	Two-Classes = 100%
[53] Multi-Classes = 100%	TWD	Statistical Features	No	KNN	Multi-Classes = 100% 99.33%
[54]	DWT	Statistical Features	N/A	SVM	Two-Classes = 97.97%
[55] Multi-Classes = 98%	IMFs	AmE	DESA	RF	Multi-Classes = 98% Two-Classes = 99.41%
[56]	DoG	LBP and Histogram Features	No	SVM	Multi-Classes = 98.80% 99.12%
[57]	GST	SVD Feature	No	RF	97.78%
[58]	DCT	HE and ARMA Model	No	LSTM	96%
[59]	DWT	Feature Extraction	No	N/A	99.26%
[60]	--	ApEn and RQA	No	N/A	95%
[61]	WT	Approximate Entropy, LLE, Correlation Dimension	FRBS	N/A	99%
[62]	Clustering, Covariance Matrix	Statistical Features	Non-Parametric Tests	AB-LS-SVM	Two-Classes = 99.64%
Proposed Method	TQWT	Statistical + Frequency + Fractal and Entropy Features	Proposed Convolutional RNN (CNN–RNN)		Multi-Classes = 99.71%

**Table 9:** Comparison of the proposed method with other related works on the Fribourg dataset.

Works	Preprocessing	Feature Extraction	Feature Selection	Classification	Accuracy
[63]	Filtering	ApEn, SampEn, PE, PFuzzy	--	SVM	95.3%
[64]	DWT	Energy, Entropy, STD, Mean	--	SVM	99.26%
[65]	FFT	--	--	CNN	92%
[66]	NA	DWT, DESA, Temporal and Spatial Averaging	Feature Aggregation	RF, Logistic, SVM	95%
[67]	WPT	Relative Amplitude, PSD, PMRS	--	weighted ELM	--
[68]	Time and Frequency Domain	--	--	CNN	--
[69]	Filtering, CSA	Linear and Non-Linear Features	--	SVM	96.8%
[70]	WT	Maximum, Minimum, Mean, STD	Bag-of-Words	SVM	--
[71]	Filtering	--	--	LSTM	97.75%
[72]	FFT, Filtering	--	--	Integer-Net	93.2%
[73]	Filtering	Different Features	--	SVM	97.5%
[74]	Filtering, HADTFD	TF-Flux, TF-Entropy, TF-Flatness	Spatial Averaging	Linear	98.56%
[75]	DWT	Uniform 1 D-LBP	--	Different Methods	95.33%
[76]	--	Linear and Non-Linear Features	Krill Herd Algorithm	Proposed Method	98.9%
Proposed Method	TQWT	Statistical + Frequency + Fractal and Entropy Features	Proposed Convolutional RNN (CNN–RNN)		99.13

## Data Availability

Not applicable.
